# Improvement of students’ communication skills through targeted training and the use of simulated patients in dental education—a prospective cohort study

**DOI:** 10.1186/s12909-024-05818-z

**Published:** 2024-07-30

**Authors:** Anna Bock, Nina Wagenknecht, Philipp Winnand, Marie Sophie Katz, Mark Ooms, Marius Heitzer, Frank Hölzle, Ali Modabber

**Affiliations:** Department of Oral and Maxillofacial Surgery, University Hospital RWTH Aachen, Pauwelsstrasse 30, D-52074 Aachen, Germany

**Keywords:** Dental education, Communication training, Simulation patients, Communication skills

## Abstract

**Background:**

Good communication between patients and practitioners is essential, especially during dental procedures, as these treatments are often associated with increased nervousness and anxiety. The aim of this study was to investigate, implement and evaluate a concept for communication skills training by using targeted training in combination with simulation patients in dental education.

**Methods:**

Students (*n* = 34) were assigned to four small groups receiving targeted training consisting of two parts. A lecture about the theoretical basics of communication skills and two practical sessions with simulation patients. During this training, one of the students performed the conversation with the patient. Immediately after self-assessment was obtained, the simulation patient, the remaining students and the lecturer provided feedback. Additionally, anonymous surveys were administered to the students at the beginning of the semester, immediately after the training and at the end of the course.

**Results:**

The students rated the learning of communication skills as important for later professional life at all times. After targeted training followed by subsequent use in simulated patients, there was a significant improvement in communication skills (*p* < 0.001). The number of open-ended questions asked to patients after attending the course significantly increased (*p* = 0.0245). The communication training was considered useful, especially in small groups.

**Conclusion:**

The implementation of targeted training with subsequent use in simulated patients significantly contributed to the students’ improvement in communication skills. The concept offers a good opportunity to better prepare students for interaction with patients, both in their studies and in their upcoming professional lives.

**Supplementary Information:**

The online version contains supplementary material available at 10.1186/s12909-024-05818-z.

## Introduction

For more than half a century, dental treatments have been available that are painless and largely shielded from other sensations. Nevertheless, many people develop strong fears and anxiety toward dental treatments. [1] To alleviate anxiety, adequate communication plays a crucial role that leads to competent clinical care. [2] Hamasaki and colleagues found that patients who viewed communication with their dentist positively had better outcomes in terms of satisfaction and lower anxiety than those who viewed communication with their dentist less positively. [3, 4] Communication between the dental practitioner and patient that creates a bond of understanding, trust and confidence may be the key to manage anxiety. [5]

Clinical communication involves complex situations influenced by diverse factors. The aim of clinical communication is to establish a connection between the patient’s requirements and preferences and the clinician’s expertise, abilities, and actions. This collaborative effort ensures that both parties work together to achieve therapeutic objectives and are content with the results of the treatment. Interpersonal exchange in this context should be patient-centered. Therefore, clinicians need to integrate communication strategies and techniques with a collection of valuable personality traits or soft skills. [6] The American Board of Orthodontics adopted 4 clinical domains to analyze patient-centered communication skills: data gathering and diagnosis, treatment objectives and planning, treatment implementation and management, critical analysis and outcome assessment. [7, 8] By paying attention to these domains and providing good communication in the relationship between patients and dentists, anxiety can be significantly minimized. [9, 10]

Current dental education is aware of the significance of empathy, socioemotional competence, and effective clinical communication. Therefore, educational concepts have changed a lot over the past decades putting these advanced skills on the same level as other core dental skills [5, 11, 12] To ensure that postgraduates are adequately trained in communication skills, these skills should be practiced and deepened in dental school through targeted training. [13] In the past, studies have shown that successful patient communication is learnable. [14, 15] For several years, simulation patients or standardized patients (actors who are trained to portray patients in clinical scenarios) have been used to provide training that incorporates the realism of clinical settings. [16, 17] These encounters have proven to be sufficient and therefore are strongly recommended teaching approaches. [18, 19] Especially in medical education, this approach has been proven to be an effective method for improving communication skills. In dental education, only a few studies assessing communication skills by simulation patients have been described. [20]

Therefore, the aim of this study was to investigate, implement and evaluate a concept for communication skills training by using targeted training in combination with simulation patients in dental education.

## Methods

### Study design

The study was performed during the summer of 2022. At the beginning of the term during the first mandatory appointment, all students in the oral and maxillofacial surgery internships (3rd, 4th and 5th year students) were invited to take part voluntarily. Students’ decisions were not influenced by the instructors and students were informed that their decision would have no impact on their grade. All invited students agreed to participate (*n* = 50). Written informed consent was obtained from each participant. All methods were carried out in accordance with relevant guidelines and regulations. Students were excluded if their attendance at the course was not completed or if no oral or maxillofacial surgery internships had taken place before (to successfully complete their studies in dentistry, students have to successfully attend the internship during their 3rd, 4th and 5th year). The students (*n* = 50) were assigned to four small groups alphabetically. The alphabetically sorted list of names was numbered one after the other from 1 to 4, so that there were 2 groups with 13 students and 2 groups with 12 students.

Within these groups, they received targeted training consisting of two parts. The first part was a lecture about the theoretical basics of communication skills, including a structured conversation process, personal approach, informed decisions and professional appearance. The second part consisted of two practical sessions with simulation patients. Overall, 4 clinical vignettes were designed to represent common clinical situations facing a dentist/oral surgeon (Table 1). The clinical examples are based on real circumstances and are intended to represent different scenarios to test various communication skills. The actors who played the simulated patients had been previously trained on the clinical case. During the training, one of the students performed the conversation with the simulation patient. Immediately after the students gave a self-assessment, subsequently they received feedback from the simulation patient, the remaining students and the lecturer. The feedback mechanism all participants were supposed to use was the sandwich technique, two positive statements surrounding a middle statement that could be perceived as negative. [21] The targeted training was integrated into an internship program at the beginning of the term, so participants had the opportunity to deepen and practice their communication skills until the end of the internship.

**Table 1:** Clinical vignettes

Scenario	Reason for consultation	Anamnese	Interactive learning goals
1	Tooth extraction with risk factors	An elderly person comes because their dentist wants 4 teeth extracted. Due to the preexisting conditions, the dentist or doctor will want this to be done in a clinic. The patient cannot say exactly why he/she should be treated in a clinic now and he/she does not know exactly his/her medication: “I trust my family doctor”. By taking a specific anamnesis, the risk factors for tooth extraction must now be determined and then a plan for the extraction must be carried out. On their own, the person hardly tells us anything about your preexisting conditions.	- Building a sustainable doctor-patient relationship - Establishing a conversation with the patient so that relevant information can be obtained - The patient should understand why their previous illnesses are relevant
2	Sialolithiasis	A patient comes to the outpatient clinic because he has been suffering from pain on the right side below the lower jaw for approximately 2 days and has also noticed an ever-increasing swelling there since this morning. He had already been to his dentist, but he could not find any cause of the swelling on the teeth. The swelling hurts significantly to the touch (and is located on the right below the mandibular bone in the soft tissue). When eating, the pain gets worse. She is concerned about the swelling, but she can swallow and breathe normally.	- Building a sustainable doctor-patient relationship - Reassure the patient as she is very worried - Patients should feel that they are in good hands
3	Eagle-syndrom	A patient has been suffering from pain in the neck area on the right side for about two years. He/she has already been to the dentist, family doctor, ENT doctor, neurologist and orthopedist several times, but they were unable to help. The patient is very desperate in view of the frequent visits to the doctor, which have not been able to provide an explanation for the symptoms and have not helped so far, and is now hoping to find out a cause and solution to the existing symptoms.	- Building a sustainable doctor-patient relationship - Calming the unsettled patient - Patients should feel well received
4	Augmentation and implantation	The patient comes to the hospital because the dentist has issued a referral for bone augmentation and subsequent implant placement. He/she has no remaining teeth in her lower or upper jaw. The dentures hold well, but there are difficulties when eating. The patient has a number of risk factors for implant treatment: smokes around 20–30 cigarettes a day, type 2 diabetes with an HbA1c of 12 and poor oral hygiene and prosthesis care.	- Building a sustainable doctor-patient relationship - Calming an upset patient - The patient should leave the conversation feeling that the decision made is the best for them

All methods were carried out in accordance with the relevant guidelines and regulations. The local Ethics Commission approved the study (EK 394/21).

### Measurements

Overall, 3 anonymous surveys were administered to the students at 3 different time points. At the beginning of the internship, immediately after the targeted training and at the end of the term. Immediately after the training, the survey served to evaluate the importance of the communication training itself in terms of its learning effect, small group sizes, feedback, motivation and teaching quality. The surveys at the beginning and end of the internship included general data, and aspects such as the importance of communication training, self-assessment and application to patients were surveyed. In addition, the integration of a few medical terms, empathetic behavior and ‘open-ended’ questions were evaluated (see supplementary material). Since all the participating students had previously participated in the internship before the targeted communication training was introduced, the first survey was also intended to evaluate the traditional format of the internship. All the surveys used 10-point Likert scales ranging from 1 = totally agree/very good to 10 = totally disagree/very bad. The surveys were distributed and collected in hard copies to the students at the beginning/end of the class.

In addition, the lecturers assessed the participants’ skills in communicating with the simulation patients on an evaluation sheet according to a mini-clinical evaluation exercise. Therefore, all aspects were rated on a 9-point scale ranging from 1 = insecure performance to 9 = surpassed/experienced (see supplementary material). To calibrate the lecturers’ evaluations on the communication skills, a training session was conducted before the start of the term. The surveys were handed to the participants in the lecture hall in paper format. Immediately after the dissemination of the surveys, students had to fill out the questionnaire and hand them back. Incomplete surveys dropped out. The reliability and validity of the surveys were checked by the course instructors before the study.

### Statistics

The obtained data were arranged using MS Office Excel 2019^®^ (Microsoft Corporation, Redmond, Washington, USA). Statistical analyses were performed using GraphPad Prism 6 software (GraphPad Software, San Diego, California, USA). The Wilcoxon signed rank test was used to compare responses within the groups. The Mann‒Whitney U test was used to compare the survey results at the beginning and the end of the semester. *P* ≤ 0.05 was considered to indicate statistical significance. Interrater reliability was tested using Kendall’s coefficient of concordance (Kendall’s W).

## Results

### Participants

Out of all assigned students (*n* = 50), there were 16 dropouts due to incomplete participation of the internship (*n* = 2) or incomplete surveys (*n* = 14). Of all successful participants (*n* = 34), 26 were women and 8 were men. Two participants were younger than 22 years old, 22 were between 22 and 25 years old, and 10 were older than 25 years. 15 participants were in their 4th year and 19 in their 5th year of the studies.

### Assessment of communication and self-assessment

**Fig. 1:**
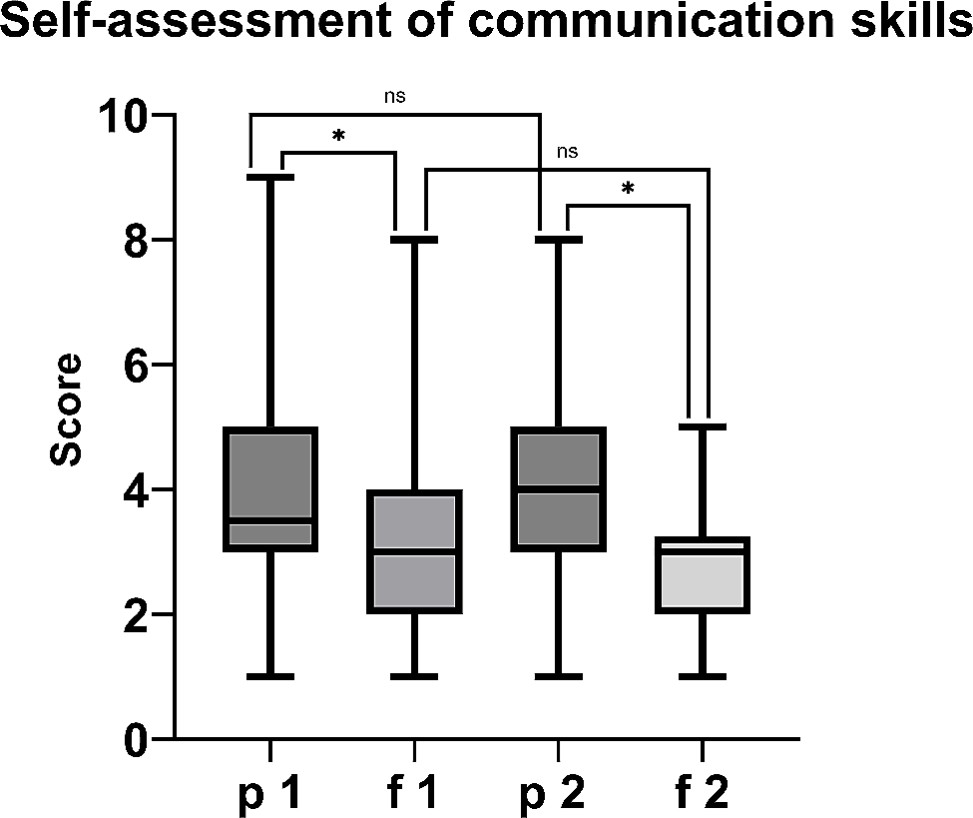
Comparison of self-assessments regarding communication skills before (p = previous) and after (f = following) the training in the first and second surveys

The results of the communication skills assessment and self-assessment are shown in Table 2. Overall, the participants rated the importance of learning communication skills during studies and later professional life as very important before and after the training. Moreover, there was no significant difference (*p* > 0.05). The participants stated that the course significantly contributed to the improvement of their communication skills after attending the training (*p* = 0.0127). Before implementing the targeted training, the students had to evaluate their former development of communication skills through the traditional internship. They stated that the traditional internship had already had a significant impact on their communication skills (*p* < 0.001). After attending the targeted training at the end of the term, they stated that the internship had already had a significant impact on their communication skills (*p* < 0.001). Comparing the first and third surveys, the participants rated their communication skills slightly better after implementing the targeted training, although the difference was not significant (*p* > 0.05). The results are shown in Fig. 1.

**Table 2:** Results of the surveys at the beginning and the end of the internship. The ranging is from 1 equaling “very important/totally agree/very good” to 10 equaling “Not important at all/Totally disagree/Very bad”

	Beginning of the internship Median (Interquartile Range)	End of the internship Median (Interquartile Range)
How do you assess the importance of learning communication skills with patients during your studies?	1 (1.75)	1 (1)
How do you assess the importance of communication skills in later professional life?	1 (0)	1 (0)
The course thus far has contributed significantly to the improvement of my communication skills with patients.	5.5 (4)	4 (2)
How do you assess your communication skills with patients before attending the course?	3.5 (2)	4 (2)
How do you rate your communication skills with patients after attending the course?	3 (2)	3 (1)
When talking to patients, I make sure to use few medical terms.	3 (2)	3 (2.75)
When talking to patients, I try to be empathetic with the patients.	1(0)	1 (1)
When talking to patients, I always try to ask “open-ended” questions.	4 (2)	3 (2)

In terms of paying more attention to using few medical terms and being empathic with patients, there was no significant difference before and after the training. Regarding the aspect of asking open-ended questions, the students stated that they paid significantly more attention to the question (*p* = 0.0245).

### Assessment of the training

Altogether, the participants rated the communication skills training and the overall teaching quality of the course very good. The results are shown in Table 3.

**Table 3:** Evaluation results of the students’ survey after the communication training. The ranging is from 1 equaling “totally agree/very good” to 10 equaling “Totally disagree/Very bad”

	Median (Interquartile Range)
Conducting communication training with simulated patients makes a lot of sense.	2.6 (2.08)
In my opinion, it makes sense to have communication training in small groups.	1.92 (1.7)
The communication training taught me important aspects of dealing with patients.	3.12 (1.88)
The feedback contributed significantly to the learning success of the communication training.	3.0 (1.96)
The communication training motivated me to apply what I had learned to the patient.	2.96 (2.03)
How would you rate the overall teaching quality of the course?	2.6 (1.6)

The lecturers had to assess the participants’ skills in communicating with the simulation patients on an evaluation sheet. They had to assess the structured approach, personal approach, fundamental decisions, and professionalism and if the student followed shared decision-making. The results are shown in Table 4. Comparing the first and the second consultation with simulation patients in all groups, the students did not show significant differences in any of the aspects (*p* > 0.05). Regarding the interrater reliability, there was some level of agreement between the raters (W = 0.635).

**Table 4:** Median and interquartile range for the assessment of student encounters with simulation patients by the lecturer. The evaluation is ranging is from 1 equaling “insecure performance” to 9 equaling “surpassed/experienced performance”

	Median (Interquartile Range)
Structured approach: - Clarifies the goals of the conversations - Follows logical sequence - Focuses on the essentials - Points out results - Divides a time	6.5 (2.25)
Personal approach: - Pays attention to the patient - Picks up on questions - Explains comprehensible - Creates an appropriate atmosphere	7.5 (2.25)
Fundamental decisions: - Applies expertise - Asks for relevant information - Available information is taken into account - Makes comprehensible decisions - Exudes professional confidence	5.5 (2.75)
Professionalism: - Stays calm - Remains authentic - Remains relaxed - Is open and interested - Knows his/her borders	8 (2)
Follows shared-decision making	6.5 (2.25)

## Discussion

Sufficient communication with patients has been largely acknowledged in recent years. It has been proven that appropriate communication with patients has a positive impact on convalescence and health outcomes, for example, pain control, psychological adjustment, and satisfaction. [22] Therefore, the aim of this study was to investigate, implement and evaluate a concept for communication skills training in dental education. To improve communication skills at our university, targeted training and encounters with simulation patients in small groups have been developed.

The survey results of this study showed that the participants were aware of the importance of communication skills training at all times. Even before attending the targeted training, they knew about the possibility of targeted communication to build a dentist-patient relationship and its subsequent influence. [6] They rated the learning of communication skills and their importance in later professional life as very important.

The evaluation of the survey prior to the implementation of the targeted training showed that the participants already stated the significant influence of the traditional course on their communication skills. The students’ prior knowledge already included the knowledge that only a few medical terms and empathic behavior should be used. Regarding the self-assessment, the results after attending the course were better than those before, regardless of the targeted skill implementation. Given these facts, it is assumed that the traditional course already positively influenced the student’s communication skills and that the students might have had a high baseline knowledge, although there was no systematic communication training before. However, the students confirmed that participation in the course involving targeted communication skills training significantly improved their skills and that the training taught them to significantly use more open-ended questions. This observation indicates the need to consider implementing these training sessions at the entry level of their clinical training, rather than waiting until the internship phase.

When evaluating the communication training immediately after attendance, they stated that the training taught important aspects of dealing with patients. In addition, the feedback contributed significantly to learning success and motivated them to apply the new learning approach. Additionally, the teaching quality itself was rated high.

In view of the assessment by the lecturers, it could be assumed that the students implemented the newly learned communication skills in the encounter medium to good. Considering this assessment, only a few students were assessed, and they were able to apply their gained knowledge. The other students only observed the conversation and participated passively. At the end of the encounter, the simulation patients, the listening students, and the lecturer provided feedback. Feedback is a crucial element of skills training because it is supposed to influence students’ motivation and capacity to learn. [23, 24] The given feedback might have influenced the students’ communication skills. In this study, the influence of the feedback was not further assessed due to the study design. Nonetheless, the impact of feedback has to be taken into account as it is considered to be a critical component of learning development and performance improvement. [25] Feedback and the associated coaching are the key to promoting students’ developmental progress towards acquiring skills. There are several established feedback models (i.e. Feedback Sandwich or Pendleton’s rules), but so far there is no empirical evidence for one being superior. [25] The latest concepts consider the feedback to be a conversation with a joint reflection of the teacher and the student. Within this conversation, trust and respect play a key role to engage a successful development. [26] In this context, it must be emphasized that training teachers to give feedback is extremely important for providing good feedback. [25, 26]

It is possible that the feedback from the first observation also influenced the performance of the listeners for the second encounter. However, this was refuted by the lecturers’ evaluation, which showed no significant difference between the first and second encounters. It would be interesting to further assess the influence of feedback on communication skills. To achieve optimal benefit from the course, it is therefore advantageous for students to be well prepared. [27]

These findings led us to conclude that the investigated concept of targeted training in combination with simulation patient encounters is a sufficient method for teaching communication skills. There are diverse methods and teaching approaches described in the literature. To date, none of the described teaching methods, either didactic or interactive, could be identified as superior to the others. Most of the described methods were valid and served their purpose [20].

As the participants noted, the performance of the encounters in small groups with simulation patients is very useful. Although small group sizes have significant advantages for students, the resource intensity of this teaching format with simulation patients must be considered. [28–30] Moreover, simulation patients face challenges in consistently depicting multiple characters within a specific setting. This makes it difficult to ensure continuity in the patient’s narrative across various scenarios and with different actors. [31] Limited time and limited resources motivate individuals to think about further solutions for adequate training in communication skills.

Due to the diverse communication styles of patients, additional research is necessary to understand the influence of different methods for learning communication skills on the patient experience. Patient experience holds significant value in shaping communication skills, and outcome measures and assessment tools in this field could be informed by patient insights. This finding provides a valuable foundation for future studies in which involving patients in assessments may guide and shape the learning process. [31]

Considering the development of modern technologies, digital learning approaches for teaching communication skills must be taken into account. For example, a review by Lee et al. stated that virtual patient simulators offer a safe and affordable learning environment. In this context, evidence-based instructional interventions combined with digital technologies can facilitate optimal use and improve learning outcomes. [32] Another study by Lin et al. suggested the positive influence of gamification. In this study, a computer role-playing game was used to teach and practice dentist-patient interactions. The study showed that the game helped to increase students’ motivation to learn behavioral issues related to communication skills. [33] In this study, the development of a digital communication skills trainer was not an option due to the lack of required resources. Nevertheless, in future studies digital options should be considered to teach communication skills.

This study has several limitations. The number of students involved in the study (*n* = 34) was small due to the limited cohort size of one study year, which makes it difficult to generalize the recommendations. Therefore, further investigations with larger sample sizes are needed. Additionally, the timing of the intervention implementation could be optimized For students it could be helpful to introduce the intervention during the early clinical phase or even to the end of the preclinical phase. This would allow students to practice their communication skills when the first start interacting with patients. Besides that, it would be interesting to investigate the long-term effect of the targeted communication skills training.

## Conclusion

In this study, the implementation of targeted training with subsequent use in simulated patients significantly contributed to the students’ improvement in communication skills. The concept offers a good opportunity to better prepare students for interaction with patients, both in their studies and in their upcoming professional lives.

## Electronic supplementary material

Below is the link to the electronic supplementary material.

Supplementary Material 1

Supplementary Material 2

Supplementary Material 3

## Data Availability

The datasets used and/or analysed during the current study available from the corresponding author on reasonable request.
