# Supplementary material for: Improvement of students’ communication skills through targeted training and the use of simulated patients in dental education—a prospective cohort study
Source: BMC Med Educ. 2024 Jul 30;24:820. doi: 10.1186/s12909-024-05818-z (PMC11290294; doi:10.1186/s12909-024-05818-z)
Supplement: Supplementary file 1 — Supplementary Material 1 [file 12909_2024_5818_MOESM1_ESM.pdf]

Evaluation beginning and end of the course

1. Demographic and general information

1.1 Gender
□ female □ male □ various

1.2 Age
□ <22 J. □ 22-25 J. □ ≥26 J.

1.3 Study year
□ 4th year □ 5th year □ 6th year

2. Communication

2.1 How do you assess the importance of learning communication skills with patients during your studies?

□1 □2 □3 □4 □5 □6 □7 □8 □9 □10
Very important Not important at all

2.2 How do you assess the importance of communication skills in later professional life?

□1 □2 □3 □4 □5 □6 □7 □8 □9 □10
Very important Not important at all

2.3 The lunchtime lecture so far has contributed significantly to the improvement of my communication skills with patients.

□1 □2 □3 □4 □5 □6 □7 □8 □9 □10
Totally agree Totally disagree

2.4 How do you assess your communication skills with patients before attending the course?

□1 □2 □3 □4 □5 □6 □7 □8 □9 □10
Very good Very bad

2.5 How do you rate your communication skills with patients after attending the course?

□1 □2 □3 □4 □5 □6 □7 □8 □9 □10
Very good Very bad

2.6 I have already had the opportunity to test my communication skills on a patient.

Yes □ No □

3. Self-assessment

3.1 When talking to patients, I make sure to use few medical terms.

□1 □2 □3 □4 □5 □6 □7 □8 □9 □10
Totally agree Totally disagree

3.2 When talking to patients, I try to be empathetic with the patients.

□1 □2 □3 □4 □5 □6 □7 □8 □9 □10
Totally agree Totally disagree

3.3 When talking to patients, I always try to ask "open-ended" questions.

□1 □2 □3 □4 □5 □6 □7 □8 □9 □10
Totally agree Totally disagree
