# Supplementary material for: Improvement of students’ communication skills through targeted training and the use of simulated patients in dental education—a prospective cohort study
Source: BMC Med Educ. 2024 Jul 30;24:820. doi: 10.1186/s12909-024-05818-z (PMC11290294; doi:10.1186/s12909-024-05818-z)
Supplement: Supplementary file 2 — Supplementary Material 2 [file 12909_2024_5818_MOESM2_ESM.pdf]

Evaluation Lecturer

Date:____

Name: ____

	Requirements according to the stage of study									
	Partially fulfilled/ insecure			fulfilled			surpassed/ experienced			unobservable / irrelevant
	1	2	3	4	5	6	7	8	9	
Structured approach:<br>- Clarifies the goals of the conversations<br>- Follows logical sequence<br>- Focuses on the essentials<br>- Points out results<br>- Divides a time	☐	☐	☐	☐	☐	☐	☐	☐	☐	☐
Personal approach:<br>- Pays attention to the patient<br>- Picks up on questions<br>- Explains comprehensible<br>- Creates an appropriate atmosphere<br>- Personal approach:	☐	☐	☐	☐	☐	☐	☐	☐	☐	☐
Fundamental decisions:<br>- Applies expertise<br>- Asks for relevant information<br>- Available information is taken into account<br>- Makes comprehensible decisions<br>- Exudes professional confidence	☐	☐	☐	☐	☐	☐	☐	☐	☐	☐
Professionalism:<br>- Stays calm<br>- Remains authentic<br>- Remains relaxed<br>- Is open and interested<br>- Knows his/her borders	☐	☐	☐	☐	☐	☐	☐	☐	☐	☐
Follows shared-decision making	☐	☐	☐	☐	☐	☐	☐	☐	☐	☐
