# Supplementary material for: Improvement of students’ communication skills through targeted training and the use of simulated patients in dental education—a prospective cohort study
Source: BMC Med Educ. 2024 Jul 30;24:820. doi: 10.1186/s12909-024-05818-z (PMC11290294; doi:10.1186/s12909-024-05818-z)
Supplement: Supplementary file 3 — Supplementary Material 3 [file 12909_2024_5818_MOESM3_ESM.pdf]

Evaluation of communication skills training

1. Demographic and general information

1.1 Gender

□ female □ male □ various

1.2 Age

□ <22 J. □ 22-25 J. □ ≥26 J.

1.3 Study year

□ 4th year □ 5th year □ 6th year

2. Communication training

2.1 Conducting communication training with simulated patients makes a lot of sense.

□1 □2 □3 □4 □5 □6 □7 □8 □9 □10
Totally agree Totally disagree

2.2 In my opinion, it makes sense to have communication training in small groups.

□1 □2 □3 □4 □5 □6 □7 □8 □9 □10
Totally agree Totally disagree

2.3 The communication training taught me important aspects of dealing with patients.

□1 □2 □3 □4 □5 □6 □7 □8 □9 □10
Totally agree Totally disagree

2.4 The feedback contributed significantly to the learning success of the communication training.

□1 □2 □3 □4 □5 □6 □7 □8 □9 □10
Totally agree Totally disagree

2.5 The communication training motivated me to apply what I had learned to the patient.

□1 □2 □3 □4 □5 □6 □7 □8 □9 □10
Totally agree Totally disagree

2.6 How would you rate the overall teaching quality of the course?

□1 □2 □3 □4 □5 □6 □7 □8 □9 □10
Very good Very bad
